# Worsening of mental health outcomes in nursing home staff during the COVID-19 pandemic in Ireland

**DOI:** 10.1371/journal.pone.0291988

**Published:** 2023-09-26

**Authors:** Conan Brady, Ellie Shackleton, Caoimhe Fenton, Orlaith Loughran, Blánaid Hayes, Martina Hennessy, Agnes Higgins, Iracema Leroi, Deirdre Shanagher, Declan M. McLoughlin

**Affiliations:** 1 Department of Psychiatry and Trinity College Institute of Neuroscience, Trinity College Dublin, St Patrick’s University Hospital, Dublin 8, Ireland; 2 Beaumont Hospital, Dublin 9, Royal College of Surgeons, Dublin 2, Ireland; 3 WellcomeTrust/Health Research Board Clinical Research Facility, Trinity College Dublin, St James’s Hospital, Dublin 8, Ireland; 4 School of Nursing and Midwifery, Trinity College Dublin, Dublin, Ireland; 5 Global Brain Health Institute, Trinity College Institute of Neuroscience, Trinity College Dublin, Dublin 2, Ireland; 6 Nursing Homes Ireland, Dublin 24, Ireland; University of Huelva: Universidad de Huelva, SPAIN

## Abstract

**Background:**

Mental health issues in nursing home staff during the COVID-19 pandemic have been significant; however, it is not known if these issues persist following widespread vaccination and easing of restrictions.

**Objective:**

To quantify the mental health of nursing home staff at different timepoints during the COVID-19 pandemic in the Republic of Ireland.

**Design/Methods:**

Two identical, online, cross-sectional, nationwide, anonymous surveys of Republic of Ireland nursing home staff at two timepoints (survey 1 (S1, n = 390): November 2020 to January 2021; survey 2 (S2, N = 229: November 2021 to February 2022) during the COVID-19 pandemic. Convenience sampling was used with staff self-selecting for participation. Methods included the World Health Organisation’s Well-Being Index (WHO-5), the Impact of Events Scale-Revised (IES-R), the Moral Injury Events Scale (MIES), two Likert-scale items regarding suicidal ideation and planning, the Work Ability Score (WAS), the Brief Coping Orientation to Problems Experienced (Brief-COPE) Scale, and a 15-item questionnaire assessing perceptions of the outbreak with one additional Likert-scale item on altruism. Descriptive analysis examined differences between staff based on their classification in one of three groups: nurses, healthcare assistants (HCA) and nonclinical staff. Pseudonymous identifiers were used to link responses across surveys.

**Results:**

An insufficient number of participants completed both surveys for linked analyses to be performed; therefore, we performed an ecological comparison between these two independent surveys. More staff reported moderate-severe post-traumatic stress symptoms (S1 45%; S2 65%), depression (S1: 39%; S2 57%), suicidal ideation (S1: 14%; S2 18%) and suicidal planning (S1: 9%; S2 15%) later in the pandemic. There was a higher degree of moral injury at S2 (S1: 20.8 standard deviation (SD) 9.1; S2: 25.7 SD (11.3)) and use of avoidant (maladaptive) coping styles at S2 (S1: 20.8 (6.3); S2 23.0 (6.3)) with no notable differences found in the use of approach (adaptive) coping styles. Staff reported more concerns at S2 regarding contracting COVID-19, social stigma, job stress, doubts about personal protective equipment and systems and processes.

**Conclusion:**

In comparison to our previous survey, mental health outcomes appear to have worsened, coping did not improve, and staff concerns, and worries appear to have increased as the pandemic progressed. Follow-up studies could help to clarify is there are any lingering problems and to assess if these issues are related to the pandemic and working conditions in nursing homes.

## Introduction

Nursing homes have endured successive waves of crises during the COVID-19 pandemic. Initially, as countries worldwide prepared for the spread of the virus, the long-term nursing home sector was an afterthought in terms of health system procurement and planning [1]. Nursing homes initially struggled to source personal and protective equipment (PPE) and staff were diverted to other, prioritised sectors, e.g., PCR testing and frontline services [2]. Subsequently, many nursing homes suffered disproportionate mortality rates globally; by mid-2020, nursing home residents accounted for 56% of COVID-19 deaths in Ireland, 40% in the USA, 47% in the UK and 40% in Italy [3–7]. This led to increased negative media scrutiny of nursing homes that was demoralising for staff [8]. Nursing home staff were forced to implement stringent regimens, including restricting visitors and group activities for residents [9]. Fortunately, vaccination rollout provided some reprieve for staff and residents and substantially reduced mortality rates [10]. However, the emergence of the Omicron variant in late 2021 added to the ongoing challenges experienced by nursing homes [9].

**Fig 1:**
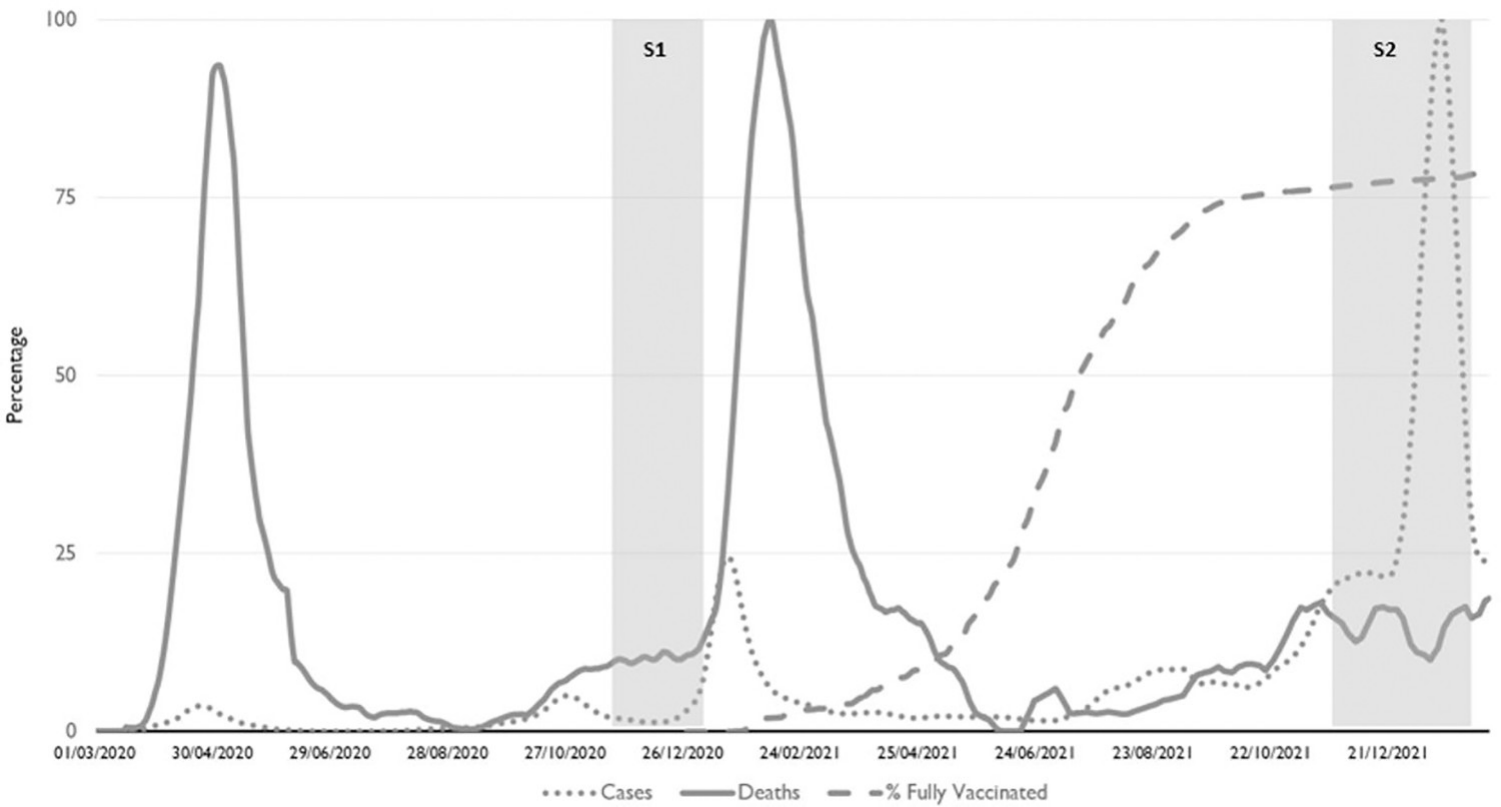
Covid-19 cases, deaths and percentage of the population fully vaccinated in the Republic of Ireland in relation to the timing of surveys 1 and 2.

These stressors seem likely to have adversely affected nursing home staff mental health [5]. While there are limited data on nursing home staff mental health prepandemic, quantitative studies indicate that there may be higher prevalences of post-traumatic stress, depression and suicidal thinking than those seen in the general population during the pandemic [11–14]. We performed an initial quantitative survey of Irish nursing home staff in late 2020/early 2021 [11]. Survey one (S1) recruited staff from 20th November 2020 to 4th January 2021 during Ireland’s third COVID-19 wave [15]. This corresponded with the escalation of cases and hospitalisations and the implementation of severe restrictions due to rapid transmission of the Alpha variant of concern (VOC) B.1.1.7 (Fig 1). This initial survey immediately preceded the rollout of COVID-19 vaccinations in the state. This study found that 45% (95%CI 40–50%) of all staff reported moderate-severe post-traumatic stress disorder symptoms; this was similar to that found in a study of Italian nursing homes (43%, 95%CI 37–49%) [16]. We found that a World Health Organisation-5 (WHO-5) wellbeing index score ≤32, indicating low mood, was reported by 38.7% (95%CI, 33.9–43.5%) of staff [11]. High levels of suicidal ideation (13.8%, 95%CI, 10.4–17.3%) and planning (9.2%, 95%CI, 6.4–12.1%) during the previous week were reported by staff.

Another area of interest during the pandemic has been the potential in various settings for moral injury in healthcare workers (HCWs), i.e., the distress experienced when one witnesses or engages in acts that contradict one’s ethical beliefs [17]. This concept arose in military mental health research; it is thought that the difficulties in providing care during a pandemic could lead to similar reactions in HCWs [18, 19]. Our initial survey indicated that there was a higher degree of moral injury in Irish nursing home staff (20.8, 95%CI 19.9–21.7) than that seen in a similar survey of UK healthcare workers in a mix of acute and mental health settings during the pandemic (15.5, 95% CI 15.1–16.0) [11, 20–22]. Additionally, in our initial survey of nursing home staff, healthcare assistants (HCAs) reported a significantly higher degree of moral injury than nonclinical (i.e., administrative and support) staff. Using an identical procedure, we performed a second survey (S2) exactly one year after our initial survey. We aimed to estimate the levels of post-traumatic stress, wellbeing, suicidal thinking, moral injury, coping styles, perceptions about the pandemic, and work ability in nursing home staff in the Republic of Ireland during the COVID-19 pandemic one year after our initial survey. We also explored if there were differences in these outcomes between professions. Finally, as these two surveys bookended Ireland’s successful vaccination programme [23], we hypothesised that this rollout of vaccination and the easing of pandemic-related restrictions might be associated with improved staff wellbeing and, correspondingly, decreased levels of post-traumatic stress, moral injury, suicidal thinking and negative occupational perceptions. Our intention was to link these two surveys using pseudonymous identifiers although we received an insufficient number of linked responses to perform meaningful analysis on these linked responses. We therefore performed an ecological comparison between these two studies.

## Methods

### 2.1 | Study design and setting

This series of two national, cross-sectional, online, anonymous surveys was approved by St Patrick’s Mental Health Services Research Ethics Committee. The target sample was all staff working in nursing homes, defined as residential facilities that provides 24-hour support and care for persons who require assistance with activities of daily living [24]. The nursing homes selected for participation were those affiliated with Nursing Homes Ireland (NHI, www.nhi.ie), the national representative body for the private and voluntary nursing home sector. NHI represents 90% of such nursing homes in the state. All 394 nursing homes on the NHI mailing-list were contacted. Convenience sampling was used with staff self-selecting for participation. Given the nature of the study, signposts to psychological supports were provided on exiting the survey. This study received ethical approval from the St Patrick’s Mental Health Services Research Ethics Committee on 18th November 2020.

### 2.2 | Recruitment and data collection

Survey 2 (S2) recruited nursing home staff from across the Republic of Ireland November 22^nd,^ 2021, to February 1^st^, 2022. This occurred in the context of a high national uptake of vaccination, rapid transmission of the Omicron VOC B.1.1.529 sublineage BA.1, declining hospitalisation rates, and a concurrent period of relative easing of lockdown restrictions. Compared to S1, this recruitment period was extended to ten weeks due to slow response uptake.

We followed a standard procedure as outlined in our previous study by contacting persons-in-charge (PICs) of all 394 NHI-affiliated nursing homes via telephone, email and post and asked them to inform their staff about the survey [11]. The survey was also advertised via social media. Data were collected online using Qualtrics Core XM (Qualtrics, USA). This software platform estimated that the survey would take approximately twenty minutes to complete. Participant information was provided, and written informed consent obtained, at survey beginning. Consent was unwitnessed as it was an anonymous survey. Minors were not included. Participation was voluntary. PICs were also asked to complete an online anonymous form on Google Forms (Google, USA) giving a simple breakdown of staff numbers in their nursing home by role.

At the end of the survey, staff were provided with an opportunity to create a unique, anonymous identifier that could be used to link their responses across surveys, with the intention that analyses could be performed on these linked responses.

### 2.3 | Measures

Identical information was collected for both surveys [11]. Briefly, basic demographic information was recorded along with level of exposure to COVID-19. Participants were asked if they had a previous diagnosed physical or mental illness prior to survey commencement. With respect to their personal history of COVID-19 infection, participants were asked if they believed that they had contracted COVID-19 irrespective of whether they had tested positive. The Impact of Event Scale-Revised (IES-R) was used for assessing post-traumatic stress symptoms during the previous seven days; this is a 22-item measure with three subscales corresponding to the main symptom domains of PTSD (hyperarousal, intrusion and avoidance). A cut-off of ≥26 indicated the presence of moderate-severe symptoms [25]. Wellbeing (and, correspondingly, mood) was assessed with the World Health Organisation’s Well-Being Index (WHO-5). This is a self-rated five-item measure that asks about wellbeing during the previous two weeks; a score of 21–32 suggests low mood and a score ≤20 suggests likely depression [26]. Two Likert scale items, derived from the Columbia Suicide Severity Rating Scale (C-SSRS), were used to appraise suicidal ideation and planning during the previous week. Responses were dichotomised depending on presence/absence of suicidal ideation or planning [27].

Adapted for healthcare staff during the COVID-19 pandemic, the Moral Injury Events Scale (MIES) was used to assess moral injury; this is a 9-item scale originally developed to appraise moral injury in military personnel [17]. Staff were asked if they agreed with statements about moral injury during the COVID-19 pandemic. The scale has three domains: “Perceived transgressions by self” (relating to acts that staff have committed that violate their own moral code), “Perceived transgressions by others” (acts of omission where staff believed they witnessed others act in ways that violated their moral code) and “Betrayal” (i.e., perceived betrayal by previously trustworthy leaders).

The Brief Coping Orientation to Problems Experienced (Brief-COPE) Scale was used to appraise staff adaptive (approach; range 12–48) and maladaptive (avoidance; range 12–48) coping styles; staff were asked to identify which coping responses they had used during the pandemic [28]. This scale also includes items for humour and religion.

We included a 15-item questionnaire adapted from a study assessing perceptions of HCWs during the severe acute respiratory syndrome (SARS) outbreak [29]. This comprised three items for each of the following perceptions: health fear, social isolation, doubts about protective equipment, dissatisfaction with infection control-related systems and processes, and job stress [30]. Items were scored from one to six; higher scores indicating greater levels of dissatisfaction. We included an additional Likert scale item assessing altruistic acceptance of risk: “Because I wanted to help COVID-19 patients, I was willing to accept the risks involved.” This was rated one to six, with higher scores indicating higher levels of altruism. There is previous evidence that altruism may mediate the psychological impact of epidemics on HCWs [31].

The Work Ability Score (WAS), derived from the Work Ability Index (WAI), is an occupational health instrument for appraising staff perceptions of work ability and identifying those needing support [32]. Participants are asked to rate their present ability to cope with work demands compared to their lifetime best on a scale of 1–10, with 10 being their lifetime best; scores ≤5 suggest insufficient perceived work ability.

### 2.4 | Statistical analysis

Surveys were not considered completed unless participants completed all measures as detailed above. Incomplete surveys were excluded from analysis. Data were analysed in SPSS 26 (IBM, USA) and Excel (Microsoft, USA). Using a 95% confidence interval with a 5% margin of error, we estimated a minimum sample size of 360 based on a previous study that reported 39% of nursing home staff during the COVID-19 pandemic scored ≥26 on the IES-R [16]. Our primary descriptive analysis of each survey examined differences between staff based on their classification in one of three groups: nurses, healthcare assistants (HCA) and nonclinical staff. The groups were further categorised depending on WHO-5, IES-R and WAS cut-off scores and the presence/absence of suicidal ideation/planning. Chi square tests were used to analyse categorical variables and one-way ANOVAs for means. Post-hoc analyses were performed for significant between-group differences with a Bonferroni correction applied. Significance level was set at 0.05; we did not adjust for multiple testing. Data are reported as means (standard deviation) and proportions (percentages with 95% confidence intervals) as appropriate.

## Results

### 3.1 Survey two (S2) descriptive analyses

#### 3.1.1 | S2 study participants

PICs from 42 of 394 nursing homes (10.75%) provided information on staff breakdown (Supplementary Table 1 in S1 File). The total number of staff working in these 42 nursing homes was 2,421. In total, 494 surveys were commenced and of these, 229 participants completed all sections and were included for analysis giving a response rate of 9.5% i.e., 229 of the 2,421 staff in participating nursing homes. These comprised 75 nurses, 100 HCAs and 54 non-clinical staff, representing respectively 15.6%, 8.0% and 7.8% of each occupational group in these participating nursing homes. Survey participants’ geographical distribution, corresponding to regional populations, is also shown in Supplementary Table I in S1 File.

Demographic characteristics of participants in S2 are summarised in Table 1. Most nursing home staff were female (86.5%), lived with their family (78.6%) and reported white ethnicity (89.1%). Most staff had no pre-existing physical (57.6%) or mental health conditions (70.7%). There were significant differences between nurses, HCAs and nonclinical staff in their living arrangements (*p* = 0.03), years of experience (*p<*0.01) and history of mental illness (*p* = 0.02). Nurses were significantly more likely to live with roommates (z = 2.6; Supplementary Table 2 in S1 File) and HCAs were significantly more likely to report other accommodation arrangements (z = 2.1; Supplementary Table 2 in S1 File). Nurses were significantly more likely to have >10 years’ experience (z = 6.0, Supplementary Table 3 in S1 File). HCAs were significantly more likely to have <10 years’ experience (z = 4.5). Nurses were less likely to have a history of mental illness (z = -2.0) and nonclinical staff were more likely to report a history of mental illness (z = 2.6, Supplementary Table 4 in S1 File).

**Table 1:** Demographic characteristics of nursing home staff, by role (Survey 2).

	Total	Nurses	HCAs	Nonclinical	Chi Square	
	*n* (%)	*n* (%)	*n* (%)	*n* (%)	χ^2^	*p* value
Total	229 (100%)	75 (32.8%)	100 (43.7%)	54 (23.6%)		
Age (years)						
≤ 30	28 (12.2%)	3 (4.0%)	16 (16.0%)	9 (16.7%)		
31–50	114 (49.8%)	43 (57.3%)	47 (47.0%)	24 (44.4%)		
≥ 51	87 (38.0%)	29 (38.7%)	37 (37.0%)	21 (38.9%)	8.369*	0.077
Gender						
Female	198 (86.5%)	69 (92.0%)	87 (87.0%)	42 (77.8%)		
Male	31 (13.5%)	6 (8.0%)	13 (13.0%)	12 (22.2%)		
Non-binary / Prefer not to say	0 (0.0%)	0 (0.0%)	0 (0.0%)	0 (0.0%)	5.469	0.065
Living Arrangements						
Alone	25 (10.9%)	9 (12.0%)	12 (12.0%)	4 (7.4%)		
With family	180 (78.6%)	59 (78.7%)	75 (75.0%)	46 (85.2%)		
With roommates	8 (3.5%)	6 (8.0%)	2 (2.0%)	0 (0.0%)		
Other	16 (7.0%)	1 (1.3%)	11 (11.0%)	4 (7.4%)	13.035*	0.031
Ethnicity						
Asian/Asian Irish	7 (3.1%)	4 (5.3%)	1 (1.0%)	2 (3.7%)		
Black/Black Irish	6 (2.6%)	2 (2.7%)	4 (4.0%)	0 (0.0%)		
Mixed race	2 (0.9%)	0 (0.0%)	2 (2.0%)	0 (0.0%)		
Other	1 (0.4%)	0 (0.0%)	1 (1.0%)	0 (0.0%		
SE Asian/SE Asian Irish	5 (2.2%)	3 (4.0%)	2 (2.0%)	0 (0.0%)		
White—Irish/British/Other	204 (89.1%)	65 (86.7%)	88 (88.0%)	51 (94.4%)		
Prefer not to say	4 (1.7%)	1 (1.3%)	2 (2.0%)	1 (1.9%)	2.167*	0.342
Years of experience						
< 5 years	76 (33.2%)	8 (10.7%)	45 (45.0%)	23 (42.6%)		
5–10 years	53 (23.1%)	13 (17.3%)	28 (28.0%)	12 (22.2%)		
>10 years	100 (43.7%)	54 (72.0%)	27 (27.0%)	19 (35.2%)	40.28	< 0.001
Physical Illness—Pre-existing**						
Cancer	2 (0.9%)	2 (2.6%)	0 (0.0%)	0 (0.0%)		
Cardiovascular Disease	47 (20.5%)	16 (21.3%)	22 (22.0%)	9 (16.7%)		
Immunosuppression	4 (1.7%)	1 (1.3%)	2 (2.0%)	1 (1.9%)		
Metabolic Disease	32 (14.0%)	9 (12.0%)	15 (15.0%)	8 (14.8%)		
Respiratory Disease	21 (9.2%)	6 (8.0%)	8 (8.0%)	7 (13.0%)		
Other	12 (5.2%)	5 (6.7%)	4 (4.0%)	3 (4.7%)		
None	132 (57.6%)	43 (57.3%)	56 (56.0%)	33 (61.1%)	0.380^†^	0.845
Mental Illness—Pre-existing**						
Anxiety disorder	44 (19.2%)	8 (10.7%)	20 (20.0%)	16 (29.6%)		
Mood disorder	40 (17.4%)	11 (14.7%)	13 (13.0%)	16 (29.6%)		
Other	8 (3.4%)	1 (1.3%)	3 (3.0%)	4 (7.4%)		
None	162 (70.7%)	59 (78.7%)	72 (72.0%)	31 (57.4%)	8.051^†^	0.018

HCAs: Healthcare Assistants. SE Asian: Southeast Asian.

*Fisher’s exact test.

**Respondents could pick multiple answers.

***Dichotomised for analysis (“White” and “Non-White”).

^†^Dichotomised for analysis (presence or absence of a pre-existing condition).

#### 3.1.2 | Exposure to COVID-19 at S2

Nursing home staff experience of COVID-19 exposure in S2 is presented in Table 2. More than half of staff reported having quarantined (52.4%). A majority reported no history of COVID-19 infection (59.0%). Of those who had contracted COVID-19, 8.8% reported having symptoms for ≥9 weeks. 42.6% reported not having fully recovered. A minority of nursing home staff reported no experience of caring for residents with COVID-19 (24.9%). 94.8% reported contact with COVID-19 infected acquaintances. There were significant differences between groups in their exposure to COVID-19 positive residents (*p*<0.001). Nurses were significantly less likely to have had no contact with COVID-19 positive residents (z = -2.7; Supplementary Table 5 in S1 File) and nonclinical staff were significantly more likely to have had no contact (z = -2.3). Initial analysis showed a significant difference between groups in terms of their history of self-quarantine (*p =* 0.046); nonclinical staff were more likely to have quarantined (z = 2.5 Supplementary Table 6 in S1 File).

**Table 2:** Nursing home staff exposure to COVID-19, by role (Survey 2).

	Total	Nurses	HCAs	Nonclinical	Chi-square	
	*n* (%)	*n* (%)	*n* (%)	*n* (%)	χ^2^	*p* value
Total	229 (100%)	75 (32.8%)	100 (43.7%)	54 (23.6%)		
Number of COVID-19 positive residents personally attended to						
None	57 (24.9%)	7 (9.3%)	26 (26.0%)	24 (44.4%)		
1–10	81 (35.4%)	24 (32.0%)	41 (41.0%)	16 (29.6%)		
11–20	31 (13.5%)	14 (18.7%)	11 (11.0%)	6 (11.1%)		
21–40	39 (17.0%)	17 (22.7%)	15 (15.0%)	7 (13.0%)		
> 40	21 (9.2%)	13 (17.3%)	7 (7.0%)	1 (1.9%)	30.728*	< 0.001
Previously self-quarantined						
Yes	120 (52.4%)	38 (50.7%)	46 (46.0%)	36 (66.7%)		
No	109 (47.6%)	37 (49.3%)	54 (54.0%)	18 (33.3%)	6.139	0.046
Previous COVID-19 infection						
No	135 (59.0%)	42 (56.0%)	65 (65.0%)	28 (51.9%)		
Yes	94 (41.0%)	33 (44.0%)	35 (35.0%)	26 (48.1%)	2.907	0.234
Symptom severity (*n* = 94)						
No symptoms	14 (14.9%)	6 (18.2%)	6 (17.1%)	2 (7.7%)		
Mild/Moderate	73 (77.7%)	24 (72.7%)	27 (77.1%)	22 (84.6%)		
Severe illness	7 (7.4%)	3 (9.1%)	2 (5.7%)	2 (7.7%)	10.525*	0.089
Symptom duration (weeks; *n* = 80)						
≤ 4	62 (77.5%)	19 (70.4%)	23 (79.3%)	20 (83.3%)		
5–8	11 (13.8%)	3 (11.1%)	4 (13.8%)	4 (16.7%)		
≥ 9	7 (8.8%)	5 (18.5%)	2 (6.9%)	0 (0.0%)	7.551*	0.089
Fully recovered (*n* = 94)						
Yes	54 (57.4%)	21 (63.6%)	18 (51.4%)	15 (57.7%)		
No	40 (42.6%)	12 (36.4%)	17 (48.6%)	11 (42.3%)	1.036	0.586
Exposure to COVID-19 positive acquaintances**						
Colleagues/Acquaintances	180 (78.6%)	67 (89.3%)	73 (73.0%)	40 (74.1%)		
Close friends	118 (51.5%)	43 (57.3%)	53 (53.0%)	22 (40.7%)		
Housemates	110 (48.0%)	38 (50.7%)	45 (45.0%)	27 (50.0%)		
Immediate family	9 (3.9%)	2 (2.7%)	2 (2.0%)	5 (9.3%)		
No contact	13 (5.2%)	1 (1.3%)	6 (6.0%)	6 (11.1%)	5.611*	0.051
Acquaintances hospitalised (*n* = 214)						
Yes	76 (35.5%)	33 (44.6%)	31 (33.7%)	12 (25.0%)		
No	138 (64.4%)	41 (55.4%)	61 (66.3%)	36 (75.0%)	5.114	0.078
Acquaintances died (*n* = 214)						
Yes	51 (23.8%)	22 (29.7%)	18 (24.3%)	11 (22.9%)		
No	163 (76.2%)	52 (70.3%)	74 (75.7%)	37 (77.1%)	2.363	0.321

HCAs: Healthcare Assistants.

*Fisher’s exact test.

**Participants could select multiple answers.

***Dichotomised to contact and non-contact for analysis.

#### 3.1.3 | Mental health measures at S2

Mental health outcomes at S2 are summarised in Table 3. The prevalence of all staff meeting the threshold for moderate-severe PTSD symptoms was 65.1% (95%CI 58.9–71.3%). There was no significant difference between roles (*p* = 0.592) in terms of prevalence of moderate to severe symptoms or on total IES-R mean (32.5, standard deviation (SD) 18.2; *p =* 0.525) or subdomain means.

**Table 3:** Nursing home staff mental health outcomes, by role (Survey 2).

	Total	Nurses	HCAs	Nonclinical	Chi-square	
	*n* = 229	*n* = 75	*n* = 100	*n* = 54	χ^2^	*p*
IES-R 22 moderate/severe, % (95% CI)*	65 (59–71)	67 (56–77)	67 (58–76)	59 (46–72)	1.050	0.592
WHO-5*						
Low mood, % (95% CI)	57 (50–63)	56 (45–67)	55 (45–65)	61 (48–74)	0.560	0.760
Likely depression, % (95% CI)	37 (31–43)	43 (32–54)	31 (22–40)	41 (28–54)	2.897	0.239
Suicidal ideation, % (95% CI)**	24 (18–29)	25 (15–35)	20 (12–28)	28 (16–40)	1.367	0.514
Suicidal planning, % (95% CI)**	15 (10–19)	16 (8–24)	11 (5–17)	20 (10–31)	2.552	0.287
Insufficient work ability, % (95% CI)*	41 (34–47)	49 (38–61)	34 (25–43)	41 (28–54)	4.178	0.125
					One-way ANOVA	
IES-R, Mean (SD)					*F*	*P*
Total	32.5 (18.2)	34.4 (18.0)	31.8 (16.5)	31.1 (21.5)	0.645	0.525
Avoidance	11.8 (7.0)	12.5 (7.0)	11.6 (6.6)	11.1 (7.8)	0.679	0.508
Hyperarousal	8.7 (5.8)	8.8 (5.4)	8.5 (5.3)	9.1 (7.1)	0.193	0.824
Intrusion	11.9 (7.1)	13.1 (7.1)	11.6 (6.6)	10.9 (7.9)	1.701	0.185
MIES, Mean (SD)						
Total	25.7 (11.3)	23.7 (10.8)	27.4 (11.3)	25.3 (11.8)	2.264	0.106
Transgression—others	6.7 (3.3)	6.0 (3.3)	7.3 (3.1)	6.7 (3.3)	3.351	0.032
Transgression—self	9.2 (5.4)	8.5 (5.3)	9.8 (5.3)	9.0 (5.7)	1.291	0.279
Betrayal	9.7 (4.7)	9.2 (4.6)	10.2 (4.6)	9.6 (5.0)	0.999	0.370
Brief-COPE, Mean (SD)						
Avoidant	23.0 (6.3)	23.2 (6.0)	22.7 (6.3)	23.2 (6.8)	0.193	0.825
Approach	28.4 (7.4)	30.1 (6.9)	27.6 (7.6)	27.5 (7.3)	3.184	0.043
Religion	3.7 (2.0)	3.8 (2.0)	3.7 (2.0)	3.6 (1.8)	0.236	0.790
Humour	3.3 (1.7)	3.4 (1.7)	3.3 (1.7)	3.4 (1.8)	0.300	0.741
COVID-19 perceptions, Mean (SD)						
Health fear	4.8 (1.1)	4.9 (1.1)	4.8 (1.2)	4.6 (1.2)	0.634	0.531
Social isolation/avoidance	3.7 (1.2)	3.8 (1.1)	3.7 (1.2)	3.7 (1.0)	0.091	0.913
Job Stress	4.7 (1.1)	4.9 (1.0)	4.6 (1.1)	4.8 (1.2)	1.695	0.186
Doubts about protection	2.9 (0.9)	2.7 (0.8)	3.0 (1.0)	3.1 (0.9)	2.142	0.120
Dissatisfaction with system/processes	3.7 (1.1)	3.5 (1.1)	3.8 (1.2)	3.8 (1.1)	2.629	0.074
Altruism perception, Mean (SD)	4.9 (1.3)	5.1 (1.2)	4.8 (1.3)	4.8 (1.2)	1.371	0.256

HCAs: Healthcare assistants. SD: Standard deviation. 95% CI: 95% Confidence Interval. WHO-5: World Health Organisation-Five Wellbeing Index: maximum of 100; cut-off of 32 or more indicates normal wellbeing. IES-R: Impact of events scale revised (22 items); cut-off of 26 or more indicates moderate to severe symptoms of post-traumatic stress. Work Ability Score: maximum of 10; cut-off of 6 or more indicates sufficient perceived work ability. MIES: Moral Injury Events Scale. Higher scores denote higher intensity of moral injury. Brief-COPE: abbreviated version of the COPE (Coping Orientation to Problems Experienced) Inventory. Higher scores indicate higher utilisation of this coping style. Perceptions of health fear, social isolation and avoidance, job stress, dissatisfaction with system/processes, doubts about protection and altruism: Higher scores indicate increased identification with each subdomain.

*Item dichotomised for analysis using cut-off score.

**Items are dichotomised for analysis (any suicidal ideation/planning vs none).

The proportion of staff reporting a WHO-5 score ≤32, indicating low mood, was 56.8% (95%CI 50.4–63.2%). Scores consistent with major depression (WHO-5 ≤20) were reported by 37.1% (95%CI 30.8–43.4%) with no significant differences between groups. Suicidal ideation over the previous week was reported by 23.6% (95%CI 18.1–29.1%) of staff and 14.8% (95%CI 10.2–19.4%) reported suicidal planning, with no differences between groups. Insufficient work ability was reported by 40.6% (95%CI 34.2%-47.0%).

The Moral Injury Events Scale (MIES) mean score for the total group was 25.7 (11.3); of the subdomains, the mean “Transgression by others” score was 6.7 (3.3); “Transgression by self” mean was 9.2 (5.4); and the “Betrayal” mean was 9.7 (4.7). There was no significant difference in the overall MIES score between groups, but a significant difference was noted between groups on the “Transgression by others” subscale (*p =* 0.028). On this measure, HCAs reported a significantly higher degree of moral injury than nursing staff (mean difference (MD) = 1.3, standard error (SE) = 0.5, *p* = 0.028; Supplementary Table 7 in S1 File).

#### 3.1.4 | Coping styles, perceptions and altruism at S2

The groups differed in the use of an approach (adaptive) coping style (*p =* 0.043). There were no significant differences found between groups on this measure on post-hoc analysis (Supplementary Table 8 in S1 File). There were no differences between groups in their use of an avoidant (maladaptive) coping style, religion or humour.

On average, staff broadly agreed with statements regarding fear of contracting COVID-19 and job-related stress. To a slightly lesser extent, they also agreed with statements indicating a degree of concern about social stigma regarding their work and about the systems and processes in place for the pandemic. They expressed less concern about infection protection measures (i.e., facemasks, eye-shields and handwashing). They agreed with an altruistic statement about accepting risks involved in caring for residents with COVID-19. There were no significant differences between groups regarding these perceptions.

## Discussion

### 4.1 Main findings

The findings of this study add to the growing body of evidence that nursing home staff report poor mental health outcomes during the pandemic [9]. The major findings of this study are the remarkably high prevalences of symptoms of PTSD, depression, moral injury, suicidal thinking at a time in a vulnerable cohort at a time when pandemic-related conditions were easing.

### 4.2 Strengths and weaknesses

Strengths of this study include the large number of validated measures used and the consistent application of recruitment and analytical methods across two surveys at similar times of the year in the same occupational population. Additionally, in the intervening time there were clear shifts in the societal impact of the pandemic in the Republic of Ireland due to the successful rollout of COVID-19 vaccinations and changes in restrictions. This provides some insight as to whether there may have been changes in nursing home staff mental health over this time period.

The first limitation of this study is it was not possible to carry out a longitudinal comparison as only one participant completed both surveys. However, this study does provide a ‘snap-shot’ of the mental health experienced by nursing home staff at these two different time points during the pandemic. Another limitation of this cross-sectional study is that the sample size is relatively small compared to the estimated number of nursing home staff in Ireland. The S2 sample size was also lower than that at S1. Therefore, it is possible that selection bias could explain these poor mental health outcomes. However, the S1 prevalence for PTSD symptoms is similar to that seen in the Italian care home study where the staff response rate was 53% [16]. Thirdly, as there are no ethnicity data available for Irish nursing home staff, it was not possible to determine if survey participants are ethnically representative of the nursing home staff population. Being a HCW of ethnic minority has been shown to be a risk factor for PTSD symptoms during the pandemic [33]. However, the Nursing and Midwifery Board of Ireland (Ireland’s regulatory body for nurses) show that the proportions of nurses’ country of registration corresponds to the ethnicity of the nurse participants in the survey [34].

### 4.3 Comparison between Survey 1 (S1) and Survey 2 (S2)

#### 4.3.1 | Anonymous linkage of survey responses between surveys

A total of two participants completed both surveys S1 and S2, indicating that the two samples are almost entirely independent. As a result, we cannot test the data for significant differences in these independent sample, but we present an ecological comparison between these two methodologically identical surveys. No further analyses were performed on linked data.

#### 4.3.2 | Demographic and COVID-19 exposure comparisons between surveys

There were no notable differences noted between groups in terms of role proportions. The sample at S1 included more participants aged 30 or younger (S1: 21.8%; S2: 12.2%) and less participants aged 60 or older (S1: 30.3%; S2 38.0%). There were no differences between surveys in terms of gender, location, accommodation, ethnicity or years of experience. A notable difference was found between surveys in terms of physical health conditions, with more respondents reporting the presence of a physical health condition at S1 (S1: 67.7%; S2: 57.6%). There was no notable difference noted between surveys in terms of the presence of mental health conditions.

There were marked differences noted between S1 and S2 surveys with respect to the amount of exposure to COVID-19 experienced by staff. As expected, higher degrees of exposure were found at S2 in the amount of contact with COVID-19 positive residents, acquaintances, quarantine history, and personal infection history. Surprisingly, given that vaccination rollout had occurred in the intervening space, there were no great differences found between S1 and S2 surveys in COVID-19 symptom severity, hospitalisation with COVID-19, duration of COVID-19 symptoms, recovery from COVID-19 and the death of acquaintances from COVID-19.

#### 4.3.3 | Mental health outcomes comparisons between surveys

The results of comparative analyses of mental health outcomes, coping styles, perceptions and altruism between surveys are displayed in Table 4. At S2, every mental health measure was noted to be worse than at S1. 45% reported moderate to severe symptoms at S1; 65% met this cut-off at S2. At S2, mean scores were higher on IES-R total score as well as on each individual subdomain of avoidance, hyperarousal and intrusion. More staff reported scores consistent with low mood (S1: 39%; S2 57%), depression (S1: 39%; S2: 57%), suicidal ideation (S1: 14%; S2 18%) and suicidal planning (S1: 9%; S2 15%) later in the pandemic. MIES scores were higher at S2, indicating a higher degree of moral injury. This was true for the total MIES score as well as for each of the subdomain scores, transgression by others, transgression by self and betrayal.

**Table 4:** Mental health outcomes compared between timepoints one and two.

	S1 *n* = 390	S2 *n* = 229
IES-R 22 moderate/severe, % (95% CI)	45 (40–50)	65 (59–71)
WHO-5		
Low mood, % (95% CI)	39 (34–44)	57 (50–63)
Likely depression, % (95% CI)	20 (16–24)	37 (31–43)
Suicidal ideation, % (95% CI)	14 (10–17)	24 (18–29)
Suicidal planning, % (95% CI)	9 (6–12)	15 (10–19)
Insufficient work ability, % (95% CI)	25 (20–29)	41 (34–47)
IES-R, Mean (SD)		
Total	25.9 (17.6)	32.5 (18.2)
Avoidance	9.7 (6.8)	11.8 (7.0)
Hyperarousal	5.5 (4.7)	8.7 (5.8)
Intrusion	9.6 (6.8)	11.9 (7.1)
MIES, Mean (SD)		
Total	20.8 (9.1)	25.7 (11.3)
Transgression—others	5.9 (3.0)	6.7 (3.3)
Transgression—self	7.9 (4.8)	9.2 (5.4)
Betrayal	7.4 (4.0)	9.7 (4.7)
Brief-COPE, Mean (SD)		
Avoidant	20.8 (6.3)	23.0 (6.3)
Approach	28.8 (8.1)	28.4 (7.4)
Religion	3.7 (2.0)	3.3 (1.7)
Humour	3.4 (1.8)	3.7 (2.0)
COVID-19 perceptions, Mean (SD)		
Health fear	4.5 (1.2)	4.8 (1.1)
Social isolation/avoidance	3.5 (1.2)	3.7 (1.2)
Job Stress	4.2 (1.2)	4.7 (1.1)
Doubts about protection	1.8 (0.8)	2.9 (0.9)
Dissatisfaction with system/processes	2.2 (0.9)	3.7 (1.1)
Altruisyuuym perception, Mean (SD)	4.8 (1.3)	4.9 (1.3)

S1: Survey 1 (November 2020 to January 2021). S2: Survey 2 (November 2020 to February 2022). SD: Standard deviation. 95% CI: 95% Confidence Interval. WHO-5: World Health Organisation-Five Wellbeing Index: maximum of 100; cut-off of 32 or more indicates normal wellbeing. IES-R: Impact of events scale revised (22 items); cut-off of 26 or more indicates moderate to severe symptoms of post-traumatic stress. Work Ability Score: maximum of 10; cut-off of 6 or more indicates sufficient perceived work ability. MIES: Moral Injury Events Scale. Higher scores denote higher intensity of moral injury. Brief-COPE: abbreviated version of the COPE (Coping Orientation to Problems Experienced) Inventory. Higher scores indicate more utilisation of this coping style. Perceptions of health fear, social isolation and avoidance, job stress, dissatisfaction with system/processes, doubts about protection and altruism: Higher scores indicate increased identification with each subdomain.

### 4.3.4 | Coping styles, perceptions and altruism between surveys

Staff reported higher use of avoidant (maladaptive) coping styles at S2 (S1: 20.8 (SD 6.3); S2 23.0 (SD 6.3)). There were no notable differences found between S1 and S2 surveys in their use of approach (adaptive) coping styles, religion or humour. Surprisingly, staff report more concern about contracting COVID-19, social stigma regarding their roles, doubts about protection and COVID-19 systems and processes at S2. This is despite the vast majority of nursing home staff being vaccinated and organisations having had almost two years to implement appropriate policies and procedures [35]. Perhaps unsurprisingly, they also report significantly more job stress but similar levels of altruism. There were no differences noted in the high levels of altruism reported by staff between these two surveys.

### 4.4 Comparison with other studies

The one-week prevalence of moderate-severe PTSD symptoms in nursing home staff in the Republic of Ireland at S2 (65%, 95%CI 59–71%) is the highest reported in nursing home staff globally to date and is notably higher than we reported previously (45%, 95%CI 40–50%) [11]. The only study with serial data examining this measure in nursing home staff is from an Italian randomised controlled trial of “Self Help Plus”, a psychological intervention. That study reported a similar prevalence of PTSD to that seen at S1 in this study but found no significant difference between their pre- and post-intervention IES-R scores (z = −0.508, p = 0.306) [12]. However, the two assessments in this Italian study occurred only 14 weeks apart and were earlier in the pandemic than both of our surveys. The best estimate for a pooled prevalence of PTSD symptoms in healthcare workers during COVID-19 pandemic to date is from a meta-review of systematic reviews and lies at 18.6–56.5% (*k* = 24, *n* = 327) [33]. This range is lower than our S2 estimate with non-overlapping confidence intervals.

The WHO-5 has been demonstrated to be useful when screening for depression during the previous 14 days [26]. While our estimate at S1 lies at the top of the range for estimated global prevalence of depressive symptoms in healthcare workers (14–37%, *k* = 28, *n* = 584), the figure at S2 is now higher than both our original study and this global figure (S1: 39%, 95%CI 34–44; S2: 57%, 50–63%) [11, 33]. Concurrently with this rise in the prevalence of depression, the already high rates of suicidal thinking seen in our second survey are also now higher. Suicidal ideation (S1: 14%, 95%CI 10–17%; S2: 24%, 18–29%) and suicidal planning (S1: 9% 95%CI 6–12%; S2: 15% 95%CI 10–19) are both higher in our second survey later in the pandemic. There is only one other study to date assessing suicidal thinking in nursing home staff during the pandemic [36]. This Italian study (n = 40) found that 25% of staff were considered high risk for suicide on the Suicide Behaviours Questionnaire-Revised. Unfortunately, drawing a comparison with our findings is not possible due to the different methodologies used. There is a paucity of current evidence of suicidal thoughts and behaviour in healthcare workers during the pandemic globally, but the figures reported here at S2 are higher than those seen in all other studies internationally [37]. The degree of moral injury experienced by nursing home staff is also greatly increased in our second survey later in the pandemic (S1: 25.9 95%CI 24.1–27.6; S2: 32.5 95%CI 30.1–34.9). The same is true of each of the moral injury subdomains. There are no other quantitative data on moral injury in nursing home staff globally, but these figures at both surveys are higher than that seen in healthcare professionals across the UK during the pandemic (15.5, 95%CI 15.1–16.0) [38]. The proportion of staff reporting insufficient work ability has greatly increased from 25% (95%CI 20–29%) to 41% (95%CI 34–47%). There are no other comparative data for nursing home staff, but lower levels of insufficient work ability have been recorded in Irish doctors before the pandemic and were associated with burn-out in doctors [39].

While the outcomes at S2 tended to be worse than at S1, it is notable that many of the significant differences detected between groups of healthcare professionals in our earlier survey are not present at this later stage of the pandemic. At S2, nurses, HCAs and nonclinical staff appeared to be equally affected in terms of post-traumatic stress symptoms, low mood and depression, suicidal thinking, perceived work ability and overall moral injury. However, we have replicated the finding from S1 that HCAs have numerically higher levels of moral injury than other professionals and both surveys showed a significantly higher level of “perceived transgressions by others” in HCAs although this was in comparison with different groups (nonclinical staff at S1 and nurses at S2).

### 4.5 Implications

This study indicates that nursing home staff mental health trended negatively as the pandemic progressed despite many pandemic-related conditions improving markedly in Ireland between S1 and S2. At the time of the second survey in the Republic of Ireland, the estimate for overall uptake of a full course of vaccination in healthcare workers and those aged ≥60 years is estimated to have been nearly 100% [35]. While the second survey took place during a surge of Omicron variant-induced cases, this period was one of widespread optimism regarding the pandemic in Ireland for two reasons. Firstly, the Irish disease burden of the Omicron wave was expected to be mild. Secondly, societal restrictions at the time were relatively benign after a prolonged period of stringent lockdown [40]. Despite this, nursing home staff reported significantly worse mental health outcomes at this time when compared to an already dismal baseline from one year earlier in the pandemic.

Concerningly, in many cases the proportions of staff reporting poor mental health outcomes at S2 were substantially larger than those observed at S1. This is not easily explained by factors present in wider society: prevalences of mental health issues in the general population appear to have been stable throughout the pandemic, both in Ireland and abroad compared to prepandemic levels [13, 41]. One clue may lie in the answers provided by staff regarding their perceptions of the pandemic. While we did not include a validated questionnaire assessing work and staffing conditions specifically, the significantly higher concerns regarding nursing home infection control systems and procedures and job stress indicate that working conditions have become more difficult over time. One possibility is that this is due to the combination of high prevalence of Omicron in the community at this time; the impact that this has had on staff shortages due to ongoing mandated COVID-19 quarantining is internationally well documented [9]. A January 2022 survey of 118 nursing homes by NHI revealed that an average of 9.4 HCAs and 2.86 nurses left nursing homes in Ireland in the previous year; 80.6% of nursing homes reported it was “extremely difficult” or “impossible” to recruit HCAs; and 61.9% reported the same difficulty with nursing recruitment [42]. The impact of this problem has likely dwarfed any potential benefits of vaccination or easing of restrictions. Unfortunately, this is unlikely to abate while highly transmissible variants of COVID-19 exist.

## Supporting information

S1 File (DOCX)
