# Supplementary material for: Worsening of mental health outcomes in nursing home staff during the COVID-19 pandemic in Ireland
Source: PLoS One. 2023 Sep 26;18(9):e0291988. doi: 10.1371/journal.pone.0291988 (PMC10521981; doi:10.1371/journal.pone.0291988)
Supplement: S1 File — (DOCX) [file pone.0291988.s001.docx]

**ST 1. Survey two: number of staff in participating nursing homes, survey participants and location of participants’ nursing homes in the Republic of Ireland, by role**

**ST2. Survey two: post-hoc analysis of significant differences in accommodation between roles**

**ST3. Survey two: post-hoc analysis of significant differences in years of experience between roles**

**ST4. Survey two: post-hoc analysis of the presence of mental illness between roles**

**ST5. Survey two: post-hoc analysis of exposure to Covid-19 positive residents between roles**

**ST6. Survey two: post-hoc analysis of quarantine history between roles**

**ST7. Survey two: pairwise comparisons of the Moral Injury Events Scale “transgression by others” subscale, by role**

**ST8. Survey two: pairwise comparisons of the Brief-COPE approach subscale, by role**

**ST 1. Survey two: number of staff in participating nursing homes, survey participants and location of participants’ nursing homes in the Republic of Ireland, by role**

|  | Total |  | Nurses | HCAs | | Nonclinical | |  |
| --- | --- | --- | --- | --- | --- | --- | --- | --- |
| Total number of staff in the 42 participating nursing homes, *n* (%) | 2,421 (100%) |  | 482 (19.9%) | | 1,251 (51.7%) | | 688 (28.4%) | |
|  |  |  |  | |  | |  | |
| Survey participants, *n* (% of total number of participants) | 229 (100%) |  | 75 (32.8%) | | 100 (43.7%) | | 54 (23.6%) | |
|  |  |  |  | |  | |  | |
| Participants’ nursing home location by province*, *n* (%) |  |  |  | |  | |  | |
| Connacht (11.7%) | 28 (12.2%) |  | 13 (17.3%) | | 7 (7.0%) | | 8 (14.8%) | |
| Leinster (56.0%) | 109 (47.6%) |  | 38 (50.7%) | | 47 (47.0%) | | 24 (44.4%) | |
| Munster (27.3%) | 77 (33.6%) |  | 20 (26.7%) | | 38 (38.0%) | | 19 (35.2%) | |
| Ulster (5.0%) | 15 (6.6%) |  | 4 (5.3%) | | 8 (8.0%) | | 3 (5.6%) | |

HCAs: Healthcare Assistants. *Populations of provinces given as percentage of total population of the Republic of Ireland (4.9 million, 2020 (est.))

**ST2. Survey two: post-hoc analysis of significant differences in accommodation between roles**

|  |  |  | Role | | | Total |
| --- | --- | --- | --- | --- | --- | --- |
|  |  |  | Nurse | HCA | Nonclinical |  |
| Accommodation | Alone | Count | 9^a^ | 12^a^ | 4^a^ | 25 |
|  |  | % | 12.0% | 12.0% | 7.4% | 10.9% |
|  |  | z-score | .4 | .5 | -.9 |  |
|  | With roommates | Count | 6^a^ | 2 ^a^ | 0 ^a^ | 8 |
|  |  | % | 8.0% | 2.0% | 0.0% | 3.5% |
|  |  | Z | 2.6 | -1.1 | -1.6 |  |
|  | With my family | Count | 59^a^ | 75^a^ | 46^a^ | 180 |
|  |  | % | 78.7% | 75.0% | 85.2% | 78.6% |
|  |  | z-score | .0 | -1.2 | 1.3 |  |
|  | Other | Count | 1^a^ | 11^b^ | 4^a, b^ | 16 |
|  |  | % | 1.3% | 11.0% | 7.4% | 7.0% |
|  |  | z-score | -2.3 | 2.1 | .1 |  |
| Total |  | Count | 75 | 100 | 54 | 229 |
|  |  | % | 100.0% | 100.0% | 100.0% | 100.0% |
| Each superscript letter denotes a subset of role categories whose column proportions do not differ significantly from each other at the .05 level (Bonferroni correction applied). | | | | | | |

**ST3. Survey two: post-hoc analysis of significant differences in years of experience between roles**

|  |  |  | Role | | | Total |
| --- | --- | --- | --- | --- | --- | --- |
|  |  |  | Nurse | HCA | Nonclinical |  |
| Years’ Experience | <10 | Count | 21^a^ | 73^b^ | 35^b^ | 129 |
|  |  | % | 28.0% | 73.0% | 64.8% | 56.3% |
|  |  | z-score | -6.0 | 4.5 | 1.4 |  |
|  | ≥10 | Count | 54^a^ | 27^b^ | 19^b^ | 100 |
|  |  | % | 72.0% | 27.0% | 35.2% | 43.7% |
|  |  | z-score | 6.0 | -4.5 | -1.4 |  |
| Total |  | Count | 75 | 100 | 54 | 229 |
|  |  | % | 100.0% | 100.0% | 100.0% | 100.0% |
| Each superscript letter denotes a subset of role categories whose column proportions do not differ significantly from each other at the .05 level (Bonferroni correction applied). | | | | | | |

**ST4. Survey two: post-hoc analysis of the presence of mental illness between roles**

|  |  |  | Role | | | Total |
| --- | --- | --- | --- | --- | --- | --- |
|  |  |  | Nurse | HCA | Nonclinical |  |
| Mental health conditions: | Yes | Count | 15^a^ | 27^a,b^ | 23^b^ | 65 |
|  |  | % | 20.0% | 27.0% | 42.6% | 28.4% |
|  |  | z-score | -2.0 | -0.4 | 2.6 |  |
|  | No | Count | 60^a^ | 73^a.b^ | 31^b^ | 164 |
|  |  | % | 80.0% | 73.0% | 57.4% | 71.6% |
|  |  | z-score | 2.0 | 0.4 | -2.6 |  |
| Total |  | Count | 75 | 100 | 54 | 229 |
|  |  | % | 100.0% | 100.0% | 100.0% | 100.0% |
| Each superscript letter denotes a subset of role categories whose column proportions do not differ significantly from each other at the .05 level (Bonferroni correction applied). | | | | | | |

**ST5. Survey two: post-hoc analysis of exposure to Covid-19 positive residents between roles**

|  |  |  | Role | | | Total |
| --- | --- | --- | --- | --- | --- | --- |
|  |  |  | Nurse | HCA | Nonclinical |  |
| Exposure to Covid-19 positive residents | Definite/probably exposure | Count | 58^a^ | 53^b^ | 19^b^ |  |
|  |  | % | 77.3% | 53.0% | 35.2% | 56.8% |
|  |  | z-score | 4.4 | -1.0 | -3.7 |  |
|  | Possible exposure | Count | 13^a^ | 31^a,b^ | 22^b^ | 66 |
|  |  | % | 17.3% | 31.0% | 40.7% | 28.8% |
|  |  | z-score | -2.7 | 0.6 | 2.2 |  |
|  | No exposure | Count | 4^a^ | 16^a,b^ | 13^b^ | 33 |
|  |  | % | 5.3% | 16.0% | 24.1% | 14.4% |
|  |  | z-score | -2.7 | 0.6 | 2.3 |  |
| Total |  | Count | 75 | 100 | 54 | 229 |
|  |  | % | 100.0% | 100.0% | 100.0% | 100.0% |
| Each superscript letter denotes a subset of role categories whose column proportions do not differ significantly from each other at the .05 level (Bonferroni correction applied). | | | | | | |

**ST6. Survey two: post-hoc analysis of quarantine history between roles**

|  |  |  | Role | | | Total |
| --- | --- | --- | --- | --- | --- | --- |
|  |  |  | Nurse | HCA | Nonclinical |  |
| Quarantine History | No | Count | 37^a, b^ | 54^b^ | 18^a^ | 109 |
|  |  | % | 49.3% | 54.0% | 33.3% | 47.6% |
|  |  | z-score | .4 | 1.7 | -2.4 |  |
|  | Yes | Count | 38^a, b^ | 46^b^ | 36^a^ | 120 |
|  |  | % | 50.7% | 46.0% | 66.7% | 52.4% |
|  |  | z-score | -.4 | -1.7 | 2.4 |  |
| Total |  | Count | 75 | 100 | 54 | 229 |
|  |  | % | 100.0% | 100.0% | 100.0% | 100.0% |
| Each superscript letter denotes a subset of role categories whose column proportions do not differ significantly from each other at the .05 level (Bonferroni correction applied). | | | | | | |

**ST7. Survey two: pairwise comparisons of the Moral Injury Events Scale “transgression by others” subscale, by role**

|  | **Role 1** | **Role 2** | **MD** | **SE** | ***p*** | **95% Confidence Interval** | |
| --- | --- | --- | --- | --- | --- | --- | --- |
|  |  |  |  |  |  | **Lower** | **Upper** |
| Transgression by others MIES | Nurse | HCA | -1.27667^*^ | .49298 | .028* | -2.4397 | -.1136 |
|  |  | Nonclinical | -.72741 | .57599 | .418 | -2.0863 | .6315 |
|  | HCA | Nurse | 1.27667^*^ | .49298 | .028* | .1136 | 2.4397 |
|  |  | Nonclinical | .54926 | .54501 | .573 | -.7366 | 1.8351 |
|  | Nonclinical | Nurse | .72741 | .57599 | .418 | -.6315 | 2.0863 |
|  |  | HCA | -.54926 | .54501 | .573 | -1.8351 | .7366 |
| MD: Mean difference. SE: Standard error. *The mean difference is significant at the 0.05 level. | | | | | | | |

**ST8. Survey two: pairwise comparisons of the Brief-COPE approach subscale, by role**

|  | **Role 1** | **Role 2** | **MD** | **SE** | ***p*** | **95% Confidence Interval** | |
| --- | --- | --- | --- | --- | --- | --- | --- |
|  |  |  |  |  |  | **Lower** | **Upper** |
| Approach style | Nurse | HCA | 2.55667 | 1.11267 | .058 | -.0684 | 5.1818 |
|  |  | Nonclinical | 2.64370 | 1.30001 | .107 | -.4234 | 5.7108 |
|  | HCA | Nurse | -2.55667 | 1.11267 | .058 | -5.1818 | .0684 |
|  |  | Nonclinical | .08704 | 1.23011 | .997 | -2.8151 | 2.9892 |
|  | Nonclinical | Nurse | -2.64370 | 1.30001 | .107 | -5.7108 | .4234 |
|  |  | HCA | -.08704 | 1.23011 | .997 | -2.9892 | 2.8151 |
